# The Yield of Community-Based “Retrospective” Tuberculosis Contact Investigation in a High Burden Setting in Ethiopia

**DOI:** 10.1371/journal.pone.0160514

**Published:** 2016-08-02

**Authors:** Zewdu Gashu, Degu Jerene, Mitiku Ensermu, Dereje Habte, Muluken Melese, Nebiyu Hiruy, Endale Shibeshi, Shallo D. Hamusse, G. Nigussie, B. Girma, Yewulsew Kassie, Yared Kebede Haile, Pedro Suarez

**Affiliations:** 1 Management Sciences for Health, Help Ethiopia Address the Low Performance of Tuberculosis (HEAL TB) Project, Addis Ababa, Ethiopia; 2 Oromia regional Health Bureau, Addis Ababa, Ethiopia; 3 Amhara regional Health Bureau, Bahirdar, Ethiopia; 4 United States Agency for International Development (USAID), Addis Ababa, Ethiopia; 5 Management Sciences for Health, Center for Health Services, Arlington, Virginia, United States of America; Johns Hopkins University Bloomberg School of Public Health, UNITED STATES

## Abstract

**Objective:**

To determine the yield and determinants of retrospective TB contact investigation in selected zones in Ethiopia.

**Materials and Methods:**

This was a community-based cross-sectional study conducted during June-October 2014.Trained lay providers performed symptom screening for close contacts of index cases with all types of TB registered for anti-TB treatment within the last three years. We used logistic regression to determine factors associated with TB diagnosis among the contacts.

**Results:**

Of 272,441 close contacts of 47, 021 index cases screened, 13,886 and 2, 091 had presumptive and active TB respectively. The yield of active TB was thus 768/100, 000, contributing 25.4% of the 7,954 TB cases reported from the study zones over the study period. The yield was highest among workplace contacts (12,650/100, 000). Active TB was twice more likely among contacts whose index cases had been registered for TB treatment within the last 12 months compared with those who had been registered 24 or more months earlier (adjusted odds ratio, AOR: 1.77 95% CI 1.42–2.21). Sex or clinical type of TB in index cases was not associated with the yield. Smear negative (SS-) index cases (AOR: 1.74 955 CI 1.13–2.68), having index cases who registered for treatment within <12 months (AOR: 2.41 95% CI 1.51–3.84) and being household contact (AOR: 0.072 95% CI 0.01–0.52) were associated with the occurrence of active TB in children.

**Conclusions:**

The yield of retrospective contact investigation was about six times the case notification in the study zones, contributing a fourth of all TB cases notified over the same period. The yield was highest among workplace contacts and in those with recent past history of contact. Retrospective contact screening can serve as additional strategy to identify high risk groups not addressed through currently recommended screening approaches.

## Introduction

Despite improvements in TB prevention and control efforts worldwide, national TB control programs miss a significant proportion of TB patients in many low and middle-income settings [1]. There is also delay in diagnosing TB and initiating treatment [2–5]. About one third of all incident cases of active TB are not properly diagnosed and there is a diagnostic delay in high TB burden settings [1, 6]. This is more pronounced in population groups with poor access to health care [4]. Even when physical access to health services is not a major challenge, people fail to seek health care for TB related complaints as people infected with TB are not symptomatic during early stages of the disease [7, 8]. Therefore, active case finding strategies are needed to detect and treat patients who are not identified through the usual passive approach.

Systematic screening of close contacts of smear positive pulmonary TB (SS+) is one of the globally recommended active case finding strategies [9]. Accordingly, contact investigation is done “prospectively”, along the course of treatment of the index case [9, 10]. We previously reported our experience with implementing contact investigation among household contacts in two regions of Ethiopia indicating significant contribution of the intervention to overall TB case finding [11].

However, organizing prospective follow up of all close contacts is not adhered to because of logistical difficulties [12, 13]. Besides, earlier contact investigation studies in Ethiopia used only household contacts of SS+ index cases [11, 14]. While there is some evidence that TB among close contacts of SS-[15–17] and in contacts other than households [18, 19] is high, this has not been demonstrated in routine program settings.

We introduced a “retrospective” contact screening approach whereby all clinical types of TB cases treated in the previous three years were listed and their contacts were traced to determine if they had developed symptoms of TB. Our objective was to determine the yield of “retrospective” community-based TB contact investigation and identify factors associated with occurrence of TB among the contacts in selected six zones in Ethiopia.

## Materials and Methods

### Design and Setting

We conducted a community based cross-sectional study in six zones with population of over 14 million in Oromia and Amhara regions of Ethiopia between June-October 2014. These zones had a higher case notification rate (CNR) of more than 130 per 100,000 from 2011–2014. The two regions have implemented the DOTS strategy for the last two decades [20, 21]. The regional health bureaus of the two regions led the implementation of the study with support from the Help Ethiopia Address the Low Performance of TB (HEAL TB), a project funded by the United States Agency for International Development (USAID).

### Data Collection

We recruited and trained lay providers to do active tracing and symptomatic screening of contacts of TB index cases. These lay providers also served as data collectors. They were recruited and deployed from the same *Kebeles* (the smallest administrative unit) of their residence, for easy tracing of past TB patients and their contacts. The data collectors were high school graduates. Together with TB focal persons and health extension workers (HEWs), they received a 2-day training on the basics of symptoms of TB including the screening algorithm, data collecting tools and standard operating procedures of the study.

### Definitions

We defined an *index case* as PTB or extra-pulmonary TB (EPTB) identified within a household registered at health facilities. A *close contact* is a person who shared the same enclosed living space for one or more nights or for frequent or extended periods during the day with the index case during the 3 months before the diagnosis of TB, such as household family, co-workers sharing same enclosed workplace or neighbors [19]. If close contacts are other than household, co-worker or neighbor they were classified as “other”. The approximate dates of contacts’ last exposure to the patients were determined using the treatment initiation data of the patients [22].

We used locally-adopted and translated version of standard symptom-based screening criteria developed by the World Health Organization [9, 10]. The criteria used in adult contacts were cough, weight loss, fever and night sweating. In child contacts the criteria were cough, weight loss or failure to gain weight, reduced playfulness, fever or/and night sweating. Presumptive TB case was defined when cough or two or more of the symptoms other than cough persisted for at least two weeks [9, 10].

TB case definition was based on the standard definitions of the National TB and leprosy control program guideline of Ethiopia for the diagnosis and treatment of TB cases [23]. Accordingly, SS+ is a patient with at least two initial sputum smear examinations positive for acid fast bacilli (AFB) by direct microscopy, or one initial smear examination positive for AFB by direct microscopy and culture positive, or one initial smear examination positive for AFB by direct microscope and radiographic abnormalities consistent with active TB as determined by a clinician. SS- is a patient with symptoms suggestive of TB with at least three AFB negative sputum smear examinations, radiographic abnormalities consistent with active pulmonary TB, no response to a course of broad spectrum antibiotics and a decision by a clinician to treat with a full course of anti-TB chemotherapy. EPTB is a patient who has TB in organs other than the lungs, with at least one specimen with M. tuberculosis or histological or strong clinical evidence consistent with active EPTB, followed by a decision by a clinician to treat with a full course of tuberculosis chemotherapy.

### The Procedure for Contact Investigation

We listed all TB cases registered for TB treatment from mid-2011 to mid-2014 in the health facility registers. Using the list, data collectors visited the index cases and traced their close contacts. The lay providers used the symptom-based screening criteria to screen the contacts. Thus, contacts that fulfilled the criteria for presumptive TB were documented as screen result positive. Otherwise, they were screen result negative. Screen negative under-five children were referred for Isoniazid preventive therapy (IPT) to health facilities.

The lay providers referred presumptive TB cases to health centers using TB suspect referral slip. At health centers, sputum examination was done using Ziehl-Neelson (ZN) microscopy, the nationally recommended TB diagnostic method [23]. Presumptive TB cases gave three sputum samples, morning-spot-morning, to diagnose pulmonary TB. EPTB and clinically suspected SS− were referred to hospitals and private clinics for chest radiography and other necessary investigations for TB. Health care workers at the health centers sent back the sputum result of the suspects to the lay providers using the feedback section of the suspect referral slip. The lay providers registered the sputum result on the contact register.

The HEWs closely monitored the work of data collectors and reviewed their performance on a monthly basis. Zonal and district TB focal persons supervised the implementation of these activities as part of their routine work.

### Data Management and Analysis

We used contact registers for the registration of traced and symptomatically screened contacts. The register had the following variables: types of index TB cases, age and sex of contacts, number of contacts per index case, type of contacts, contacts screened, screening result, presence or absence of active TB, and type of TB cases identified. The register served as primary data source for the study based on which data entry template was prepared using the Cis-pro software. We exported the data to STATA for analysis. We have uploaded the minimal data set without identifier of the study participants as supporting information (S1 Dataset).

To ensure data quality, randomized blinded quality check was made. Data was also entered to excel based performance monitoring system for consistency check. In addition, each data element was run independently to identify data entry errors. Zonal and district TB focal persons supervised the data collection to ensure completeness of data. Hence, there was only 0.23–0.44% missed data. Average imputation method for age, common-point imputation for period when index cases registered for anti-TB and modal imputation for type of contacts and sex was applied to fill in the missed values [24]. There was no unique pattern in the missing data on these variables.

We used frequency, percentage and mean to describe index cases and their contacts. The yield is described using proportion and per 100, 000 of contacts with 95% confidence interval (95% CI). We used logistic regression analysis to determine factors associated with TB diagnosis among the contacts. The outcome variable, TB diagnosis, was labeled as 1 if TB was detected and 0 if no TB detected. Variables with p-value less than 0.2 in univariate analysis were included in the multivariable analysis. We conducted a subgroup analysis of child contacts <15 years to determine factors associated with cases of TB in children.

### Ethical Statement

Ethics Review Committees of Oromia and Amhara Regional Health Bureaus approved the study protocol, oral informed consent procedure and the data collection tool. Letters of permission to implement the intervention and access to TB registers were obtained from relevant authorities. Only contacts who gave oral consent to participate in this study were screened for TB. We used oral consent because the study included predominantly rural population who could not read and write. In the contacts of age less than 18 years, their parents or guardian were asked for consent. Contacts with TB diagnosis received care according to the standard practice.

## Results

### Characteristics of Index Cases and their Contacts

We included 47,021 index cases registered in the 427 health facilities of the study zones during the five month of study period. About 43% of these had been registered for anti-TB treatment before 24 months during data collection period. The rest (57.3%) initiated the treatment within 24 months of data collection period. Forty-one percent of the index cases were SS+.

Of 272,515 eligible close contacts approached, the lay workers screened 272,441(99.97%) close contacts. The proportion of screened contacts among total population in the study zones was 1.9%. The ratio of contacts to index cases was 5.8. About 43% of the contacts were identified from SS+ index cases whereas the respective 29% and 28% were from SS- and EPTB index cases. Household, neighbor, work place and other contacts constituted 63%, 11.3%, 0.6% and 25.7% respectively. About 52.5% and 64.6% of the contacts were male and adults or adolescents of age greater than 14 years, respectively (Table 1).

**Table 1:** Characteristics of index cases registered and contacts with index cases approached for screening in the six study zones, June-October 2014, Ethiopia.

Variables	Number	Percent (%)
**Index cases**		
**By type of TB**		
SS+	19235	40.9
SS-	13652	29
EPTB	14134	30.1
Total	47021	100
**By the period they registered for treatment**		
<12 months	15251	32.4
12–23 months	11678	24.8
> = 24 months	20092	42.7
**Contacts with index case registered**		
**Contacts approached by type of index cases**		
SS+	116324	42.7
SS-	78721	28.9
EPTB	77470	28.4
Total	272515	
**Type of contacts**		
House hold	170136	62.4
Neighbor	30585	11.3
Workplace	1643	0.6
Other	70151	25.7
**Contacts by sex**		
Male	143143	52.5
Female	129372	47.5
**Contacts by Age Category**		
<5 years	22655	8.3
05–14 years	73963	27.1
> = 15 years	175897	64.6
**Contacts based on the period their index cases registered for treatment**		
<12 months	89822	33
12–23 months	66669	24.5
> = 24 months	116024	42.6

### The Yield of TB Screening

**Fig 1:**
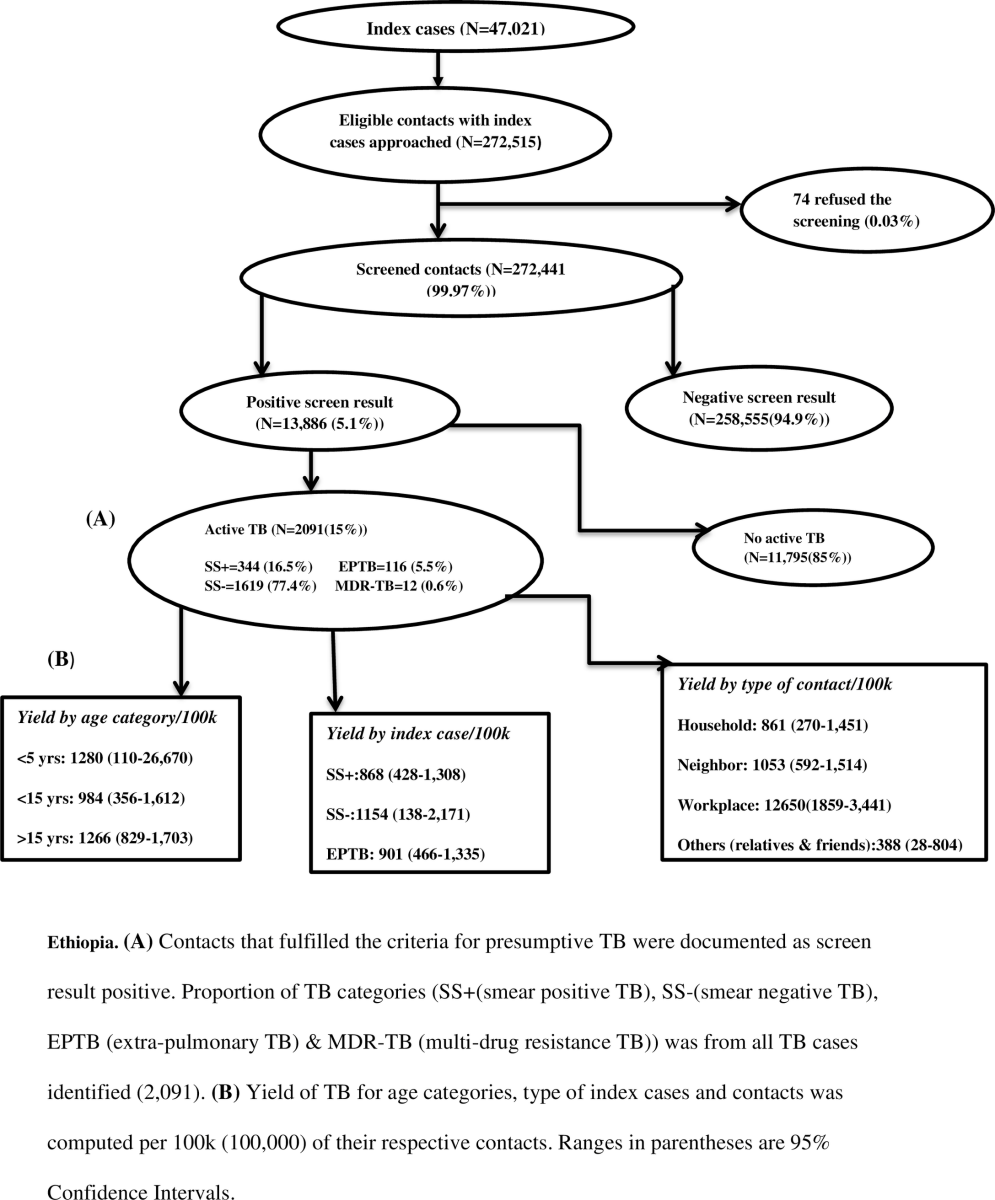
Flow diagram of screening and yield of retrospective contact investigation, June-October 2014, Ethiopia.

**Fig 2:**
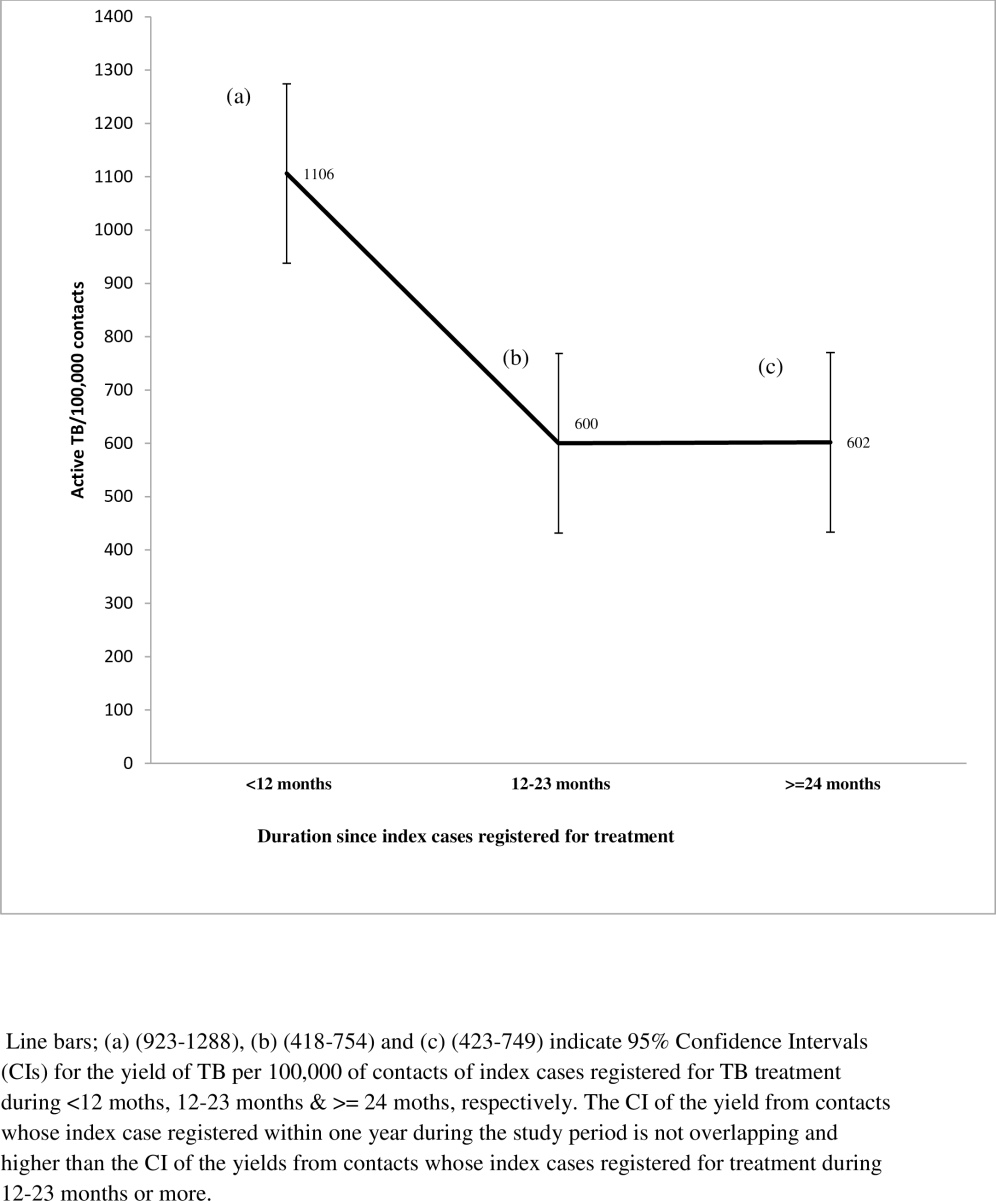
Yield per 100,000 contacts based on the time since index cases registered for TB treatment.

Of those screened, 13,886 (5.1%) and 2, 091 (0.8%) were found to have presumptive and active TB respectively. The yield of all forms of TB per 100, 000 contacts was thus 768/100, 000. Of the 2,091 active TB cases diagnosed through contact screening, 77.4% were SS- while 16.5% were SS+ cases. Active TB cases detected through the retrospective screening constituted 25.4% of the 7,954 TB cases reported in the study zones during the study period. The prevalence of SS+ among the adult contacts was 106/100,000. The proportion of SS+ among presumptive TB cases was 2.5%. TB cases detected among household contacts were 0.96%. Also, the respective yield per 100, 000 among households, neighbors and workplace contacts was 861, 1053 and 12, 650 (Fig 1). For contacts whose index cases registered for treatment < 12 months, 12–23 months and > = 24 months, the respective yield per 100, 000 contacts were 1106, 600 and 602 (Fig 2).

After adjusting for co-variates, the rate of active TB was 1.77 times higher among contacts whose index cases registered for treatment within the last 12 months than contacts that had been exposed 24 or more months earlier (AOR: 1.77 95% CI 1.42–2.21). The rate of active TB was higher in the age group of 25–34 years (AOR: 1.80 95% CI 1.2–2.62) and 35–44 years (AOR: 2.14 95% CI 1.42–3.22) as compared to under-five children. The odds of active TB cases from neighbor (AOR: 1.35, 95% CI 1.02–1.78) and workplace (AOR: 3.95: 95% CI 2.21–7.03) were significantly higher than active TB cases detected from household contacts. However, the yield from contacts of “other” category was less than the yield from household contacts (AOR: 0.13, 95% CI 0.08–0.20).There was no significant difference in yield between close contacts of SS+ index cases and those of EPTB (AOR: 0.88: 95% CI 0.69–1.13) and SS- index cases (AOR: 1.19: 95% CI 0.95–1.49) (Table 2).

**Table 2:** Factors associated with active TB in retrospective contact investigation, June–October 2014 Ethiopia.

Variables	Active TB case (%)		Active TB [1] versus non-TB[0] cases		
		COR	95% CI	AOR	95% CI
**Year index cases registered for TB treatment**					
<12 months	993 (1.4)	2.04	1.64–2.54	1.77	1.42–2.21
12-23months	400 (0.7)	0.98	0.74–1.30	0.93	0.7–1.24
> = 24 months	698 (0.7)	1			
**Type of Index cases registered**					
SS+	854 (0.9)	1			
EPTB	507 (0.7)	0.8	0.63–1.03	0.88	0.69–1.13
SS-	730 (1.1)	1.23	0.98–1.53	1.19	0.95–1.49
Age C**ategory of contacts**					
Children (<15 years)	581 (0.7)	0.68	0.54–0.84	0.55	0.44–0.69
Adult (>15 years)	1510 (1.0)	1			
**Sex of Contacts**					
Female	951 (0.74)	1			
Male	1140`(0.80)	1.15	0.95–1.40	1.12	0.92–1.37
**Type of contacts**					
House hold	1627(1.1)	1			
Neighbor	352(1.4)	1.23	0.94–1.62	1.35	1.02–1.78
Workplace	78(4.8)	4.5	2.55–7.94	3.95	2.21–7.03
Other	34(0.2)	0.14	0.09–0.22	0.13	0.08–0.20

Being contact of SS- index cases (AOR: 1.74 95% CI 1.13–2.68), having index cases who registered for treatment within <12 months (AOR: 2.41 95% CI 1.51–3.84), and being household contact (AOR: 0.072 95% CI 0.01–0.52) were factors that were significantly associated with the occurrence of active TB in children (Table 3).

**Table 3:** Determinants of active TB in children <15 years through retrospective contact screening, June-October 2014, Ethiopia.

Variables	Number (%) of active TB	Active TB [1] and no TB [0] in children <15 years of age			
		COR	95% CI	AOR	95% CI
**Type of Index cases**					
SS+	223 (0.6)	1			
SS-	242 (1.0)	1.85	1.20–2.84	1.78	1.16–2.75
EPTB	116 (0.5)	0.88	0.51–1.50	1.004	0.59–1.72
**Time index cases completed treatment**					
<12 months	313 (1.1)	2.69	1.69–4.27	2.4	1.50–3.82
12 months—23months	131 (0.6)	1.4	0.78–2.51	1.31	0.73–2.36
> = 24 months	137 (0.4)	1			
**Type of Contacts**					
Household	542 (0.8)	1			
Neighbor	32 (0.4)	0.47	0.21–1.08	0.51	0.22–1.17
Workplace and Others	7 (2.3)	0.12	0.03–0.51	0.14	0.035–0.58
**Sex of contacts**					
Female	312 (0.70)	1			
Male	269 (0.73)	0.95	0.64–1.39	Not applicable	Not applicable

## Discussion

To our knowledge, this is the first report of the yield of retrospective TB contact screening in a community setting in Ethiopia through which we were able to detect over two thousand TB cases. The yield was about six times the case notification rate in the study zones and contributed about a quarter of all notified cases over the same period. Our findings suggest that retrospective contact screening can be considered a useful strategy for identifying additional TB cases not addressed through the routinely implemented case finding strategies.

Earlier studies reported the yield of contact screening among household contacts of SS+ index cases using prospective screening approach [11, 14, 25–29]. The yield of 0.96% among household contacts in the current study is comparable with what was reported by Salinas et al [30]. On the contrary, it is lower than the yield by the prospective approaches; 2.5% in similar setting in Ethiopia [11], 6.07% in South Africa [28] and the global average of 3.1% [31]. However, the overall yield in our study is about six times the case notification rate in the study zones during the same time period. The yield among contacts whose index cases registered for TB treatment within 12 months was eight times the TB case notification in the study zones. Thus, our finding clearly highlights the need to include retrospective contact screening, at least for contacts whose index cases registered for treatment within the past one year, as one of the strategies for case detection.

The 2.5% SS+ cases among the presumptive TB cases in this study might be underestimate but still relatively higher than the corresponding rate of 1.2% in the Ethiopian National TB prevalence survey [32]. However, the 106/100,000 prevalence of SS+ among the adult contacts is much higher than the result from the TB prevalence survey in Eritrea [33] and equivalent to the prevalence of 108/100,000 in the national TB prevalence survey in Ethiopia [32].

Our study revealed a significantly higher yield of active TB among workplace contacts as compared to other types of contacts. This might be due to the fact that the index cases share the same enclosed space for longer hour and the spaces might be overcrowded and poorly ventilated coupled with the little awareness on TB prevention [34]. A study on contact screening at workplace from Portugal revealed that the yield was 8.4 TB cases per index case [19]. This suggests that there is a need to consider contact tracing beyond households especially in congregated workplaces such as schools, mining areas and prisons. Further studies should include detailed work place related variables such as employment status, hours and working conditions so as to generate more evidence on factors associated with increased risk of TB in work place contacts.

We also involved neighbor visitors of the sick index cases for contact investigation. This is because most neighbors in rural Ethiopia are relatives and genetically related to the sick. It is also part of the tradition of Ethiopian society to visit and stay with the sick while they are possibly exposed. The yield was higher at 1.4% among the neighbor contacts. Cheng et al (2015) from Uganda showed that first degree relatives’ contacts were more likely to be symptomatic for TB [18]. It was also shown by Lienherdt et al (2003) in Gambia that development of TB cases increased with first degree relatives compared with more distant and non-genetically related households [13]. In fact, it is possible that genetic factors contributed to the susceptibility to TB infection [15]. Also, Classen et al (1999) indicated the need to target contacts outside of households in high incidence TB areas to reduce TB transmission [35]. These studies from elsewhere suggest the need to consider close relatives for contact screening, and the higher yield among the neighbors in the current study suggests that retrospective contact is also a feasible strategy for contacts of neighbor and relatives.

The yield of TB among adult contacts was higher than that of child contacts, which is likely to be related with underdiagnoses among children due to diagnostic difficulties [36]. Most of the TB cases were also identified from close contacts of TB patients with SS+ which is in line with most studies [11, 15, 26, 27, 29, 30, and 37].The greater proportion of childhood TB was detected from the contacts of SS-. It could be due to the selective nature of the prospective contact screening through which contacts of SS+ cases might have already been identified and taken care of. However, it needs further clarification in future studies. The fact that SS- can contribute to TB transmission has been shown in other studies [15–17]. The strategy of screening only those in contact with SS+ cases is likely to miss about one third of infected individuals [38].

Through the retrospective contact screening approach, we also detected TB cases from close contacts of EPTB index cases. Likewise, there are studies which included EPTB as an index case during active case findings [12, 39]. Contacts of patients with EPTB were evaluated because there are possibilities of associated pulmonary TB (PTB) cases [15]. Laryngeal TB and pleural TB are EPTB but can transmit TB as well [15–22]. There is also the opportunity to identify the real index cases of the identified EPTB that failed to be detected through the routine case detection strategy. Thus, in settings where TB is highly prevalent and there is a challenge of delay in the diagnosis there are possibilities of missed TB cases in the community [40]. Seeking for contacts of EPTB could detect the undiagnosed and missed TB cases which could be the real index cases of the EPTB. These could be cases that shared other common index cases but failed to seek health service. Therefore, comprehensive contact tracing should be considered in high burden settings.

The findings in this study should be interpreted cautiously as there were some limitations. We used symptom screening and light microscopy to diagnose SS+. Hence, a chance of missing the SS+ cases cannot be ruled out [41] though our study was done at health facilities that participated in a regular AFB microscopy external quality assessment (EQA) with concordance of 95% on random blinded rechecking [42]. In other studies, using digital X-rays in addition to symptom screening and fluorescent microscopy for diagnosis could not also detect all SS+ cases individuals [32, 33]. Also, we included limited number of variables which did not allow thorough evaluation of all the potential determinants of TB among contacts. In addition, only few of the neighbor close contacts were accessed and screened as most of them did not fulfill the criteria of close contacts. However, this is the first study reporting the yield of retrospective contact screening from Ethiopia and perhaps one of a few globally [43]. The other strength of this study is the large number of contacts screened compared with earlier reports.

## Conclusions

The yield of retrospective contact screening through community-based approach was about six times the case notification in the study zones and contributed a significant proportion of all cases notified in the study districts. The risk of TB was high among contacts irrespective of the type of TB in the index case. This highlights that retrospective contact screening can be of high yield strategy among all types of index TB cases especially within one year of the registration of the index case. The yield was highest among work place contacts, suggesting the need to prioritize work place interventions for TB prevention and control. Further implementation and evaluation of retrospective contact screening should be done in similar settings to validate these findings. Such evaluations should include cost and cost-effectiveness studies.

## Supporting Information

S1 Dataset Data set for the retrospective contact investigation study. (DTA)

S1 Table The contact investigation register. (DOCX)

S1 Text Information sheet and oral informed consent form. (DOCX)
