# Supplementary material for: The Yield of Community-Based “Retrospective” Tuberculosis Contact Investigation in a High Burden Setting in Ethiopia
Source: PLoS One. 2016 Aug 2;11(8):e0160514. doi: 10.1371/journal.pone.0160514 (PMC4970728; doi:10.1371/journal.pone.0160514)
Supplement: S1 Table — (DOCX) [file pone.0160514.s002.docx]

| **Logbook of Contact tracing** | | | | | | | | | | | | | | | | | |
| --- | --- | --- | --- | --- | --- | --- | --- | --- | --- | --- | --- | --- | --- | --- | --- | --- | --- |
| Zone________Woreda___________Name of HF___________ | | | | | | | | | | | | | | | | | |
|  |  |  |  |  |  |  |  |  |  |  |  |  |  |  |  |  |  |
| 1)S No | 2)HFs name | 3) Address | 4)Full Name Including grand father | 5)TB Forms of index case | 6) Date of Anti TB started for Index case (D/M/Y) | 7) # of contacts | 8) List of contacts (Name) | 9) Sex & age of contact 1.Male2.Female | 10)Types of contact | 11)TB system. screening for contacts Result code & date | | | 12) Dx result code & date of close contacts | | | 13) Action Taken code & date at Dx | 14)Remark |
|  | Unit TB NO | Kebele/Gote | Index Name | 1)SS+ 2)SS-VE 3)EPTB |  |  |  |  | 1.HH 2.Neigh 3.Workplace 4. Other | 1st visit | 6 month visit | 12 month visit | 1st visit | 6 month visit | 12 month visit | 1.TB Rx started 2. Referred to Other HF for TB Dx 3. IPT started  4. Other |  |
|  |  | HH Number | Contact person to support Rx |  |  |  |  |  |  | 1. P 2.N | 1. P 2.N | 1. P 2.N | 1)SS+ 2)SS-VE 3)EPTB 4)MDR 5.no TB | 1)SS+ 2)SS-VE 3)EPTB 4)MDR 5.no TB | 1)SS+ 2)SS-VE 3)EPTB 4)MDR 5.no TB |  |  |
|  |  |  |  |  |  |  |  |  |  |  |  |  | 1 |  |  |  |  |
|  |  |  |  |  |  |  |  |  |  |  |  |  |  |  |  |  |  |
|  |  |  |  |  |  |  |  |  |  |  |  |  |  |  |  |  |  |
|  |  |  |  |  |  |  |  |  |  |  |  |  |  |  |  |  |  |
|  |  |  |  |  |  |  |  |  |  |  |  |  |  |  |  |  |  |
|  |  |  |  |  |  |  |  |  |  |  |  |  |  |  |  |  |  |
|  |  |  |  |  |  |  |  |  |  |  |  |  |  |  |  |  |  |
|  |  |  |  |  |  |  |  |  |  |  |  |  |  |  |  |  |  |
|  |  |  |  |  |  |  |  |  |  |  |  |  |  |  |  |  |  |
|  |  |  |  |  |  |  |  |  |  |  |  |  |  |  |  |  |  |
|  |  |  |  |  |  |  |  |  |  |  |  |  |  |  |  |  |  |
|  |  |  |  |  |  |  |  |  |  |  |  |  |  |  |  |  |  |
|  | | | |  | | | | | | | | | | |  |  |  |
