# Supplementary material for: The Yield of Community-Based “Retrospective” Tuberculosis Contact Investigation in a High Burden Setting in Ethiopia
Source: PLoS One. 2016 Aug 2;11(8):e0160514. doi: 10.1371/journal.pone.0160514 (PMC4970728; doi:10.1371/journal.pone.0160514)
Supplement: S1 Text — (DOCX) [file pone.0160514.s003.docx]

**Information Sheet and Oral Consent Form**

**Study title: The Yield of Community-based “Retrospective” Tuberculosis Contact Investigation in a High Burden Setting in Ethiopia**

My name is ----------- from Oromia/ Amhara Regional Health Bureau. Here we are interviewing individuals who have been in contact with TB patients started anti-TB treatment three years back. It is aimed to find out missed TB cases and stop TB transmission in the community. We believe that this study would help to detect TB cases and reduce TB transmission. We would like to assure you that your name will not be mentioned in the questionnaire and the information that you give us will be kept confidential and only used for research purposes. You have a full right to refuse to take part or to interrupt the interview at any time. In case you have cough complaint exceeding 2 weeks, we will refer and examine your sputum for the presence of TB at the nearby health facility. But the information that you will give us is quite useful to achieve the objective of the study and to bring change in the TB case finding. It will take 20-25 minutes to respond to the interview.

Are you willing to participate in the study?

1- Yes 2 - No

(If the answer is yes, thank the patient and put your signature. Then conduct the interview. Otherwise, the patient is not forced to participate)

I [Data Collector] confirm that I have fully explained the purpose of the study to the study participant who is consenting.

Name of Data Collector_________________________Signature___________Date:____________

Contact Person: Dr. Degu Jerene (MD, PHD), Principal Investigator, Addis Ababa,Tel: +251911546401
